# CyFi-MAP: an interactive pathway-based resource for cystic fibrosis

**DOI:** 10.1038/s41598-021-01618-3

**Published:** 2021-11-15

**Authors:** Catarina Pereira, Alexander Mazein, Carlos M. Farinha, Michael A. Gray, Karl Kunzelmann, Marek Ostaszewski, Irina Balaur, Margarida D. Amaral, Andre O. Falcao

**Affiliations:** 1 Faculty of Sciences, BioISI—Biosystems Integrative Sciences Institute, University of Lisboa, Campo Grande, 1749-016 Lisbon, Portugal; 2 LASIGE, Faculty of Sciences, University of Lisboa, Campo Grande, 1749-016 Lisbon, Portugal; 3 Luxembourg Centre for Systems Biomedicine, University of Luxembourg, 6 Avenue du Swing, 4367 Belvaux, Luxembourg; 4 CIRI UMR5308, CNRS-ENS-UCBL-INSERM, European Institute for Systems Biology and Medicine, Université de Lyon, 50 Avenue Tony Garnier, 69007 Lyon, France; 5 Biosciences Institute, University Medical School, Newcastle University, Framlington Place, Newcastle upon Tyne, NE2 4HH UK; 6 Universität Regensburg, 9147 Regensburg, Germany

**Keywords:** Systems biology, Diseases, Cellular signalling networks, Mechanisms of disease, Chloride channels, Molecular medicine

## Abstract

Cystic fibrosis (CF) is a life-threatening autosomal recessive disease caused by more than 2100 mutations in the CF transmembrane conductance regulator (CFTR) gene, generating variability in disease severity among individuals with CF sharing the same *CFTR* genotype. Systems biology can assist in the collection and visualization of CF data to extract additional biological significance and find novel therapeutic targets. Here, we present the CyFi-MAP—a disease map repository of *CFTR* molecular mechanisms and pathways involved in CF. Specifically, we represented the wild-type (wt-CFTR) and the F508del associated processes (F508del-CFTR) in separate submaps, with pathways related to protein biosynthesis, endoplasmic reticulum retention, export, activation/inactivation of channel function, and recycling/degradation after endocytosis. CyFi-MAP is an open-access resource with specific, curated and continuously updated information on CFTR-related pathways available online at https://cysticfibrosismap.github.io/. This tool was developed as a reference CF pathway data repository to be continuously updated and used worldwide in CF research.

## Introduction

Omics technologies revolutionized the way researchers generate data^1^. The integration of disease-specific data allows not only to capture knowledge at its multiple levels of organization but also to unbiasedly identify molecular features, such as phenotypes associated with complex and heterogeneous diseases—which would remain otherwise unnoticed^2,3^. The visual representation of molecular mechanisms has emerged as a powerful tool for a better understanding and analysis of specific disease-causing features. This led to the development of several pathway-based resources, such as KEGG (Kyoto Encyclopedia of Genes and Genomes)^4^, Wikipathways^5^, Reactome^6^ and MetaCore™^7–10^.

The need for dedicated knowledge maps as tools for the representation of mechanisms involved in specific diseases in a holistic form gave origin to the concept of a disease map. A disease map consists of a representation of disease mechanisms illustrating the major signalling, metabolic and regulatory pathways known to be involved in a specific disorder in an interchangeable format. The major feature of the disease map is to allow comparisons among different maps using a high-quality representation in a standardised format^11^. Furthermore, it is computer-readable, user-friendly and can be transformed into mathematical models for predictive analysis and new hypothesis generation^11^. This resource can store and display multiple layers of information—from subcellular, cellular, tissue, organ to whole organism systems—besides providing customizable levels of detail that can be used in the illustration of the biological mechanisms^12^. In the past, to further understand their mechanisms and potentially find therapeutic targets, several disease maps for complex disorders have been developed such as Parkinson’s^13^, Alzheimer’s^14^, asthma^15^, several types/forms of cancer^16^ and rheumatoid arthritis^17^.

Cystic Fibrosis (CF) is a life-shorting rare genetic disorder affecting 90,000 to 100,000 individuals worldwide, that results from over 2,100 variants in the CF transmembrane conductance regulator (*CFTR*) gene^18–20^. The CFTR protein functions as a chloride/bicarbonate channel activated through cAMP-induced phosphorylation at the apical plasma membrane (PM) of epithelial cells. This protein works in a dynamic network, interacting with multiple components and regulating a significant number of other channels^21–23^.This channel loss of function causes the organs in which it is expressed to be impacted. Specifically, in the lung *CFTR* malfunction causes severe airway dehydration and thickening of the lung mucus, leading to impaired mucociliary clearance (MCC) and, subsequently, clogging of the airways. This causes progressive loss of lung function, which is the major cause of morbidity and mortality^24,25^. Furthermore, it is important to note that *CFTR* loss of function does not occur only in CF, but also in chronic obstructive pulmonary disease (COPD)—the third main cause of death worldwide^26^—revealing the crucial role this protein plays in the airway epithelium^27^.

The most common CF-causing mutation on the *CFTR* gene is the c, which occurs in approximately 70% of people with CF worldwide^28^. F508del-*CFTR*is a misfolded unstable protein, which is mostly retained in the endoplasmic reticulum (ER) and targeted for ER-associated degradation (ERAD). However, if F508del-*CFTR* bypasses this pathway and is rescued to the PM, the protein still presents defective channel gating and low PM stability, being rapidly endocytosed and degraded^29–31^. The biogenesis and intracellular trafficking of F508del-*CFTR* protein has been extensively studied to better understand the pathways that contribute to and drive CF progression. Notwithstanding, and owing to the inherent complexity of the mechanisms and pathways, the *CFTR* dynamic network is still only partially understood^20,32–35^.

Several databases and software tools have been used in efforts to represent the known CF mechanisms through the representation of pathways covering different processes—such as MetaCore™ from Clarivate Analytics (i.e., CFTR folding and maturation), Reactome (i.e., RHO GTPases regulate *CFTR* trafficking), KEGG (i.e., ABC transporters—Homo sapiens) and Wikipathways (i.e., ABC-family proteins mediated transport)^6,8,36^. Notwithstanding, these pathway-based databases present *CFTR* data incorporated with non-related processes and proteins such as other ABC-family proteins, which although allow comparison between some members of this family, may interfere with the reading and interpretation of *CFTR* specific data. Furthermore, most are not detailed and/or updated with recent disease features, besides not being freely accessible, nor containing *CFTR* variants information or allowing comparison between a wt and mutant protein interactions, essential to understanding the progression of this disease. yFi-MAP is developed and publicly available as a complementary resource to the existing tools. Moreover, CyFi-MAP content benefits from being discussed and approved by domain experts (available on the GitHub page, section Team). The incorporation of CFTR molecular mechanisms in a single resource represented in a standardized and inter-exchangeable manner represents one of the main advantages of CyFi-MAP. This tool allows the user to: (i) follow *CFTR* pathways; (ii) acquire its functional context and subcellular localization through the visualization of the cell compartments represented in the map; (iii) interpret differences between wt-CFTR and F508del-*CFTR* where unique interactions with *CFTR* variant are highlighted.

With this goal in mind, in this work, we aimed to build a repository of the available *CFTR*-related knowledge as a disease map named CyFi-MAP. CyFi-MAP is distinctive from other *CFTR* databases by being the first CF disease map and aiming to archive molecular mechanisms and biological pathways reported to be relevant to *CFTR* in a standard and consistent way. This repository is continuously updated with careful manual annotations and is expected to expand in both molecular mechanisms and in CFTR mutations representation and to be continuously updated based on the published literature. Through the map, it is possible to visualize *CFTR* pathways in protein cycle processes described in a sequential network, resulting in a more global biological interpretation and a faster understanding of CF pathophysiology.

## Results

### Concept and features

CyFi-MAP offers a resource that accurately (i.e., adequately confirmed in the literature) and graphically illustrates *CFTR* molecular pathways in an easy-to-read manner by the scientific community. Given that CF is caused exclusively by mutations in the *CFTR* gene that alter multiple cellular functions, the CyFi-MAP information was organized according to the *CFTR* life cycle, from its biogenesis to degradation, where two sub-maps were developed: (i) one representative of wt-*CFTR* and (ii) one of F508del-*CFTR*.

**Figure 1:**
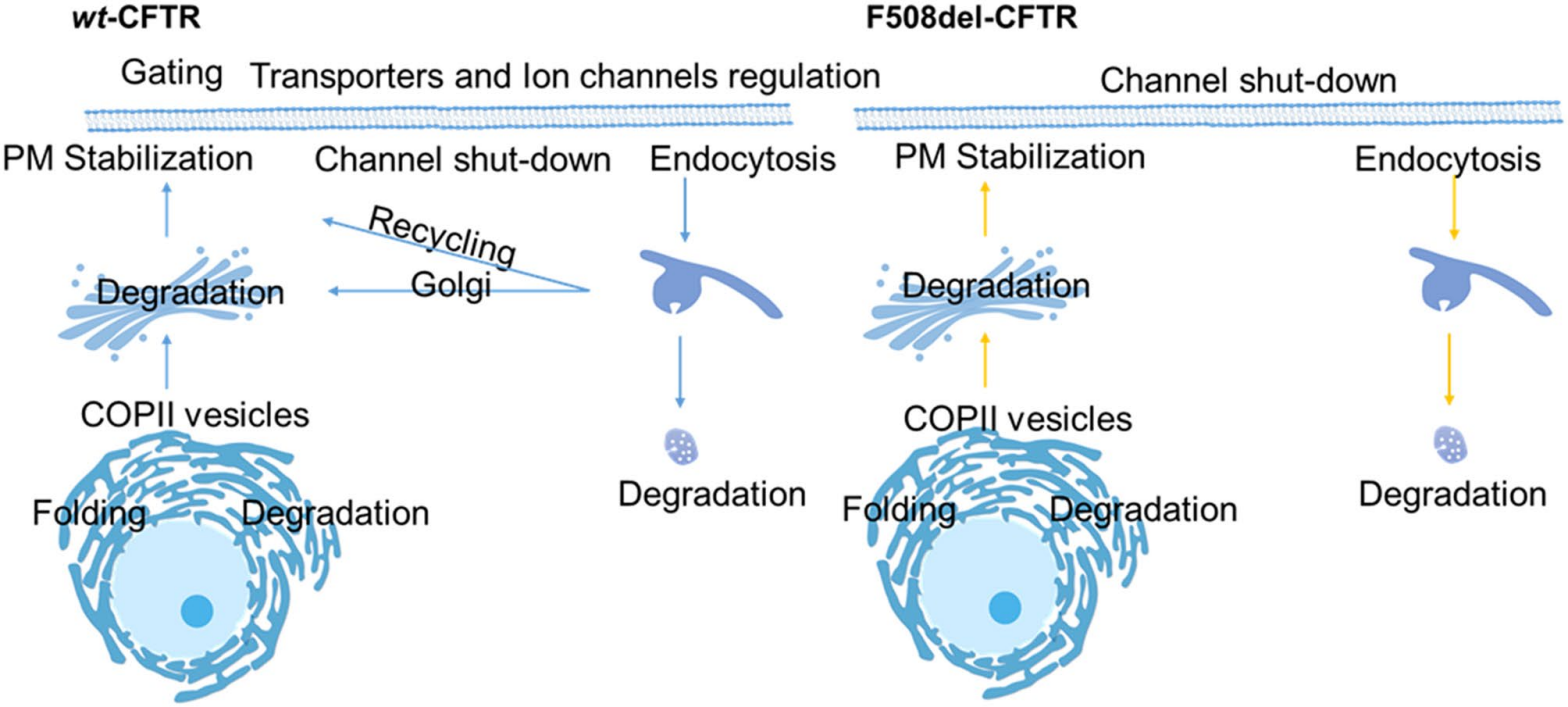
Modules available in the CyFi-MAP. wt-*CFTR* (left) and F508del-*CFTR* (right) modules include the key processes available included in the CyFi-MAP and provide a way to focus on a specific part of *CFTR* life cycle. Rescued F508del-*CFTR* (rF508del-CFTR) pathways are highlighted in yellow, indicating the processes elicited after chemical or temperature rescue of the mutant protein (see text for details).

The main differences between these submaps are given by the representation of some of the key processes side-by-side (available on the website in the Map section), in order to facilitate submap comparison at molecular mechanism level. Additionally, a scheme is also available with *CFTR* traffic pathways inside the cell where the major physical alterations (e.g., traffic impairment and mucus clogging) in wild-type vs the mutant in airway epithelial cells are depicted (Fig. S1). An overview of the key *CFTR* processes/modules included in CyFi-MAP is given to inform and guide the user to the map content (Fig. 1).

### Pathway inclusion strategy

We included information on *CFTR* interactors that was confirmed in a minimum of two published references. We focused on those that studied airway epithelial cells and used methodologies that allowed the detection of physical interaction between components (such as immunoprecipitation or nuclear magnetic resonance spectroscopy (NMR)). We also captured information on confidence and accuracy to each interaction in the map. Specifically, this step resulted in 296 research papers providing physical evidence between proteins and more than 1000 papers reviewed from PubMed (for more details on map curation see “Methods” section).

CyFi-MAP presents features that allow retrieving information visually such as (1) different types of interactions between the entities (i.e., activation, trafficking and inhibition—more details in Fig. S2 in Supplementary material), (2) the glycosylation (i.e., form B or C) and folding status (N-glycosylation) of *CFTR*, (3) the proteins that bind uniquely to the F508del-*CFTR*protein, (4) identification of eachlife cycle steps included—additional information in Supplementary material, and (5) cell organelles specific interactions represented through images, with the entities (i.e., proteins, complexes, ions, and others) adequately located in the biological compartments, differentiating between organelle lumen and the cytosol.

### CyFi-MAP navigation

#### Map availability

The source of these schemes are available at the map online repository (https://cysticfibrosismap.github.io/) and the CyFi-MAP can be accessed online and explored interactively via the Molecular Interaction NetwoRks VisuAlization (MINERVA)^37^.

#### Online and interactive navigation

**Figure 2:**
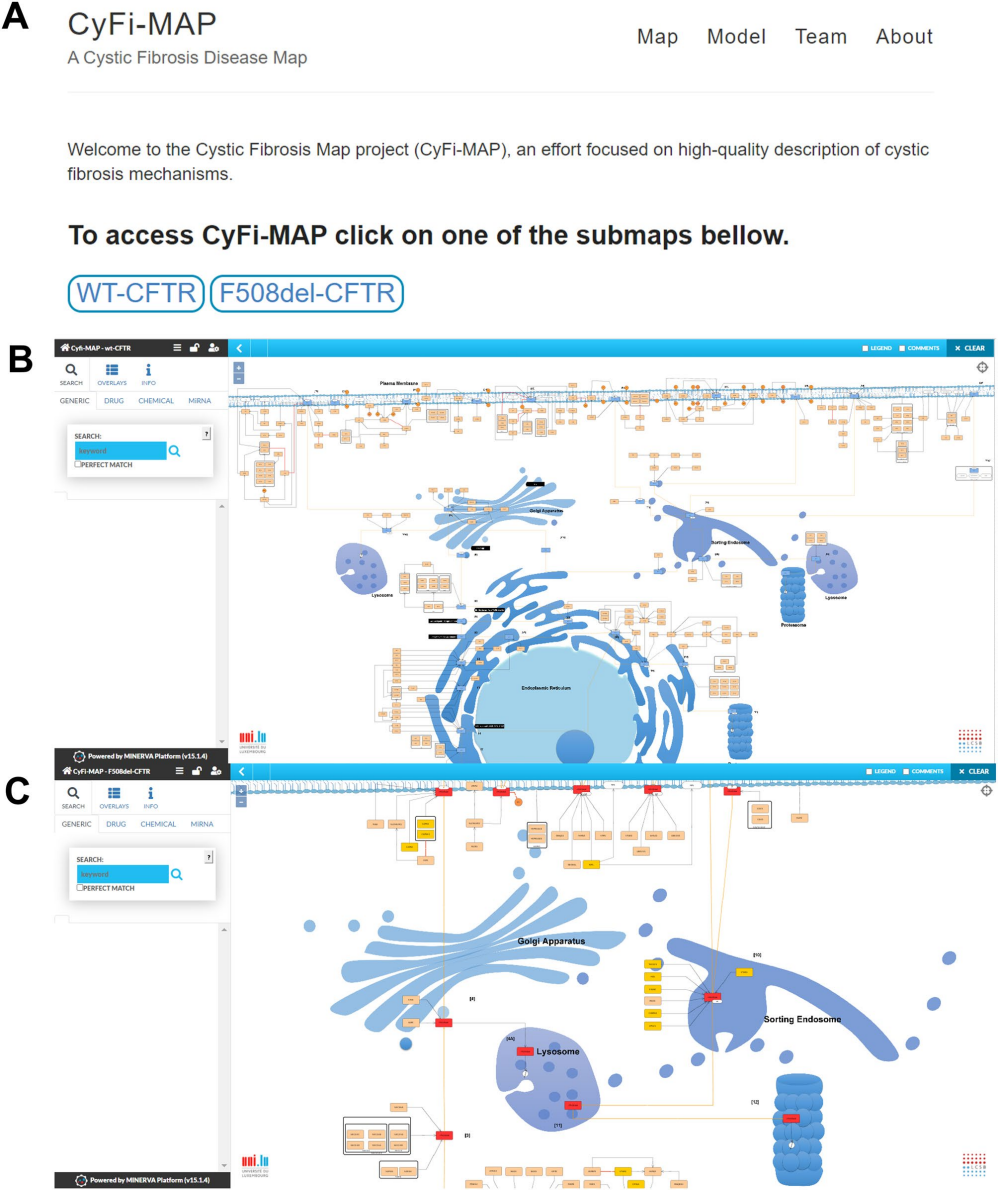
CyFi-MAP Github and MINERVA platform. In the image (**A**) is possible to see the GitHub page with the objective to contextualize this work and provide access to the interactive MINERVA platform. In image (**B**) and (**C**) are represented both submaps, wt-*CFTR* and F508del-*CFTR* respectively, where it is possible to navigate and explore the pathways and interactions included in CyFi-MAP, providing generic information about the proteins and components.

In the form of an interactive diagram via the MINERVA platform (Fig. 2), CyFi-MAP provides the capacity to easily follow *CFTR* interactions starting with its folding until degradation, with every intermediate step described (see supplementary material). The MINERVA platform allows easy navigation and exploration of the CF related molecular pathways available in CyFi-MAP. The user can also zoom in and find various details about proteins of interest (such as location of their interactions with other biological elements inside a cell, information on the protein name/alternative names and identification names in several databases such as Ensembl, human gene nomenclature, UniProt etc.; the annotation information is linked to the biological resources via direct urls). The user can also filter and extract information regarding the type of interaction through the edge colour (see Fig. S2 in Supplementary Material).

### Content of the CyFi-MAP

The progression of CF disease is driven by the deregulation of multiple cell processes due to the loss of *CFTR* function. Hence, CyFi-MAP has greatly focused on the *CFTR* proteostasis network, as it encompasses alterations occurring on all those processes. Along with the map it is possible to observe several numbers which represent each step on the *CFTR* life cycle (a brief description of these is given in supplementary material) although the order indicated is not deterministic, serving only as an indication of all the processes in which CFTR is involved. The content included in the CyFi-MAP can be divided into four mains aspects: (i) *CFTR* synthesis and production, (ii) maintenance at the PM in the functional state, (iii) traffic and (iv) degradation.

#### CFTR synthesis and production

The folding of *CFTR* is highly regulated in the ER before the protein is allowed to proceed along the secretory pathway to the PM. This information is represented in the Folding module at both submaps hence, it is possible to visualize it side-by-side in the GitHub link.

**Figure 3:**
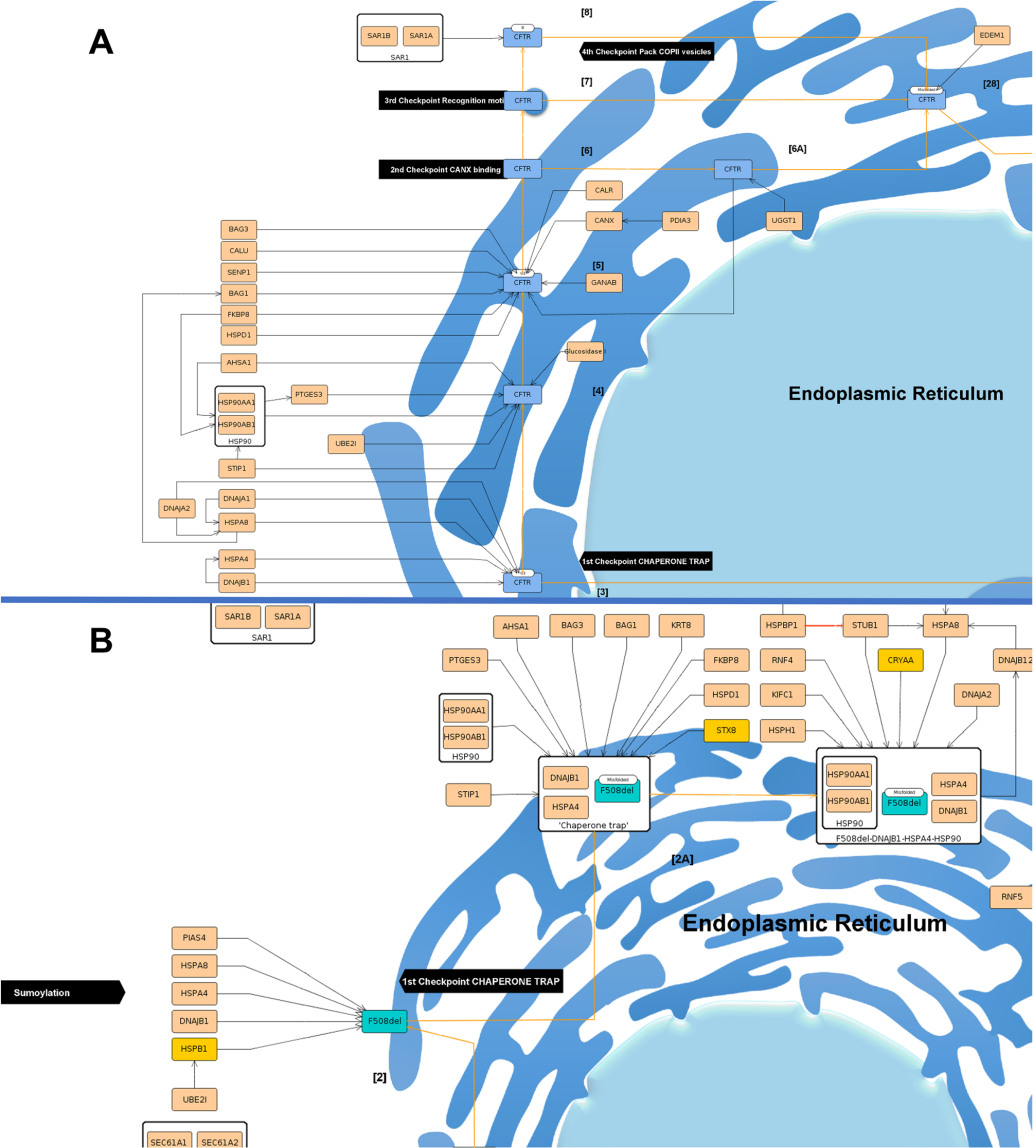
Representation of folding at ER. Wt-*CFTR* (**A**) and F508del-*CFTR* (**B**) are subjected to the sequential ERQC checkpoints, indicated in black. On the wt-*CFTR* is possible to see the four checkpoints of the *CFTR* protein (in blue). In the case of F508del-*CFTR* is possible to see a bifurcation in the pathway, where it can be targeted to degradation or rescued, where it appears red at the cytosol. It is also indicated the beginning of the sumoylation degradation pathway. The proteins are represented in beige with the orange lines indicating the movement of the protein and the black lines indicating stimulation. The white boxes containing several proteins indicate a complex. Unique interactors to F508del-*CFTR* are represented in yellow.

Beginning with thewt-*CFTR*, folding starts with the nascent *CFTR CFTR* polypeptide chain being translocated to the ER membrane (step [1]), after which the N-glycosylation occurs (step [2]), a posttranslational modification during protein synthesis in the ER that is critical for PM expression and function (Fig. 3A)^38–40^.In CyFi-MAP, the different glycosylation states of wt-*CFTR* are represented and possible to follow. N-glycosylation starts with the addition of oligosaccharide residues (glucose3-mannose9-N-acetylglucosamine2) to *CFTR* (Fig. 3A, step [2]). At this step, several chaperones and co-chaperones bind to wt-*CFTR* to assist with folding. The folding process includes at least four ER quality control (ERQC) checkpoints that are involved in assessing *CFTR* correct folding^41^.As the process consists of the subsequent trimming of glucose, they are initially identified with three glucose (G3), the first checkpoint, before binding to calnexin (*CANX*) with only one (G1), the second checkpoint (Fig. 3A, steps [3–6])^42^.

F508del-*CFTR*is mostly targeted to degradation in the first checkpoint (Fig. 3B, step [2]), hence the other ERQC checkpoints are only represented at the wt-*CFTR* submap There, is possible to follow the second checkpoint, where the protein enters the *CANX *cycle for additional rounds of refolding, the third checkpoint, where specific signals when exposed lead to ER retention, or the fourth checkpoint, with the recognition of an export motif to leave ER (Fig. 3A, step [7] and step [8])^43^. During these checkpoints, wt-*CFTR* can be recognized as misfolded and move to the degradation or achieves an incomplete glycosylated state known as form B, which allows it to proceed to Golgi. A more detailed description of these checkpoints is in the degradation chapter.

Although insertion in the membrane of ER and ERQC checkpoints that lead to degradation are identified as different steps, there is evidence that co-translational folding and degradation occur.

After *CFTR* folding has been successfully achieved, the protein is ready to proceed along the secretory pathway to the Golgi apparatus, where its oligosaccharide structure is further modified by multiple glycosylation events generating its mature form, known as C form, which will be transported to the PM^44^. F508del-*CFTR*, because is highly degraded at ER, from the moment is depicted outside this organelle acquires a dark red colour, representing a rescue protein rF508del-*CFTR*.

#### Maintenance at the plasma membrane in the functional state

After delivery to the PM, wt-*CFTR* is regulated at multiple levels namely: (1) PM stabilization, at specific PM sites; (2) activation/channel shut down, where phosphorylation/dephosphorylation cycles activate/inactivate the channels; (3) ion channels and transporters regulation, concerning the regulation of and by other PM proteins; and (4) endocytosis, in either CCVsor caveolae vesicles.

In contrast, rF508del-*CFTR* is characterized by (1) PM stabilization; (2) channel shut-down; and (3) endocytosis, with consequent degradation. The reduced number of modules at PM is representative of the instability, loss of function and accelerated endocytosis that characterize this mutated protein.

**Figure 4:**
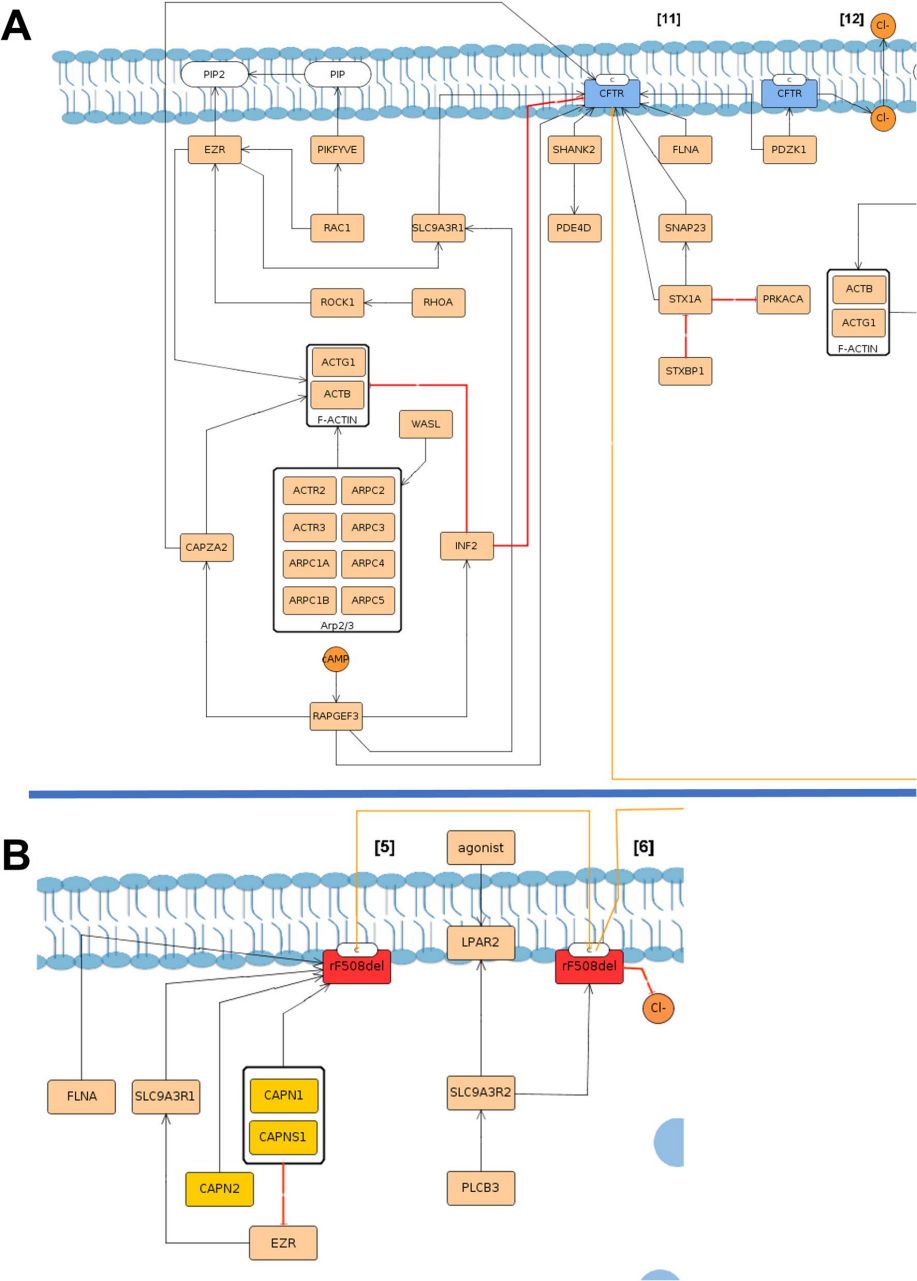
PM stabilization in wt-*CFTR* (**A**) and F508del-*CFTR* (**B**) submaps. Wt-*CFTR* represented in blue is delivered to the apical PM in the C form, as indicated at the protein, and several proteins responsible for the anchoring to the PM bind. The lines show different types of interactions, where orange lines indicate traffic, being possible to see in chloride (Cl^−^) transport across the membrane in orange. The black lines indicate binding/stimulation and the red one’s inhibition. F-actin and Arp2/3 are represented as complexes, with several proteins with a grey line separating them from the rest. In blue are represented 1-phosphatidyl1D-myo-inositol-4-phosphate (PIP) which by the action of Phosphoinositide Kinase, FYVE-Type Zinc Finger Containing (*PIKFYVE*) is converted to 1-phosphatidyl-1D-myo-inositol-4,5-bisphosphate (PIP2) with a blue line depicting synthesis. Although rF508del-*CFTR* reaches the PM, the binding to new proteins highlighted in yellow diminishes its stabilization and anchoring therefore accelerating its endocytosis and consequent degradation.

In the PM stabilization module, Postsynaptic density 95, disks large, zonula occludens-1 (PDZ) domain-containing proteins are the main characters, responsible for anchoring *CFTR* to the PM^45^. Mechanisms such as cytoskeletal activation are represented, which enable PM anchoring and tethering of wt-*CFTR* to the PM (Fig. 4A, step [11]). TherF508del-*CFTR* stabilization module includes a lower number of interactions with PDZ proteins and acquisition of new interactions when compared to the wt-*CFTR* module (Fig. 4B, step [5]).

*PDZK1* (*CAP70*) is illustrated at PM in the wt-*CFTR* submap to be able to potentiate the CFTR chloride channel activity by cluster two *CFTR* molecules (wt-*CFTR* submap, step [12])^46^ (Fig. 4A, step [12]).

**Figure 5:**
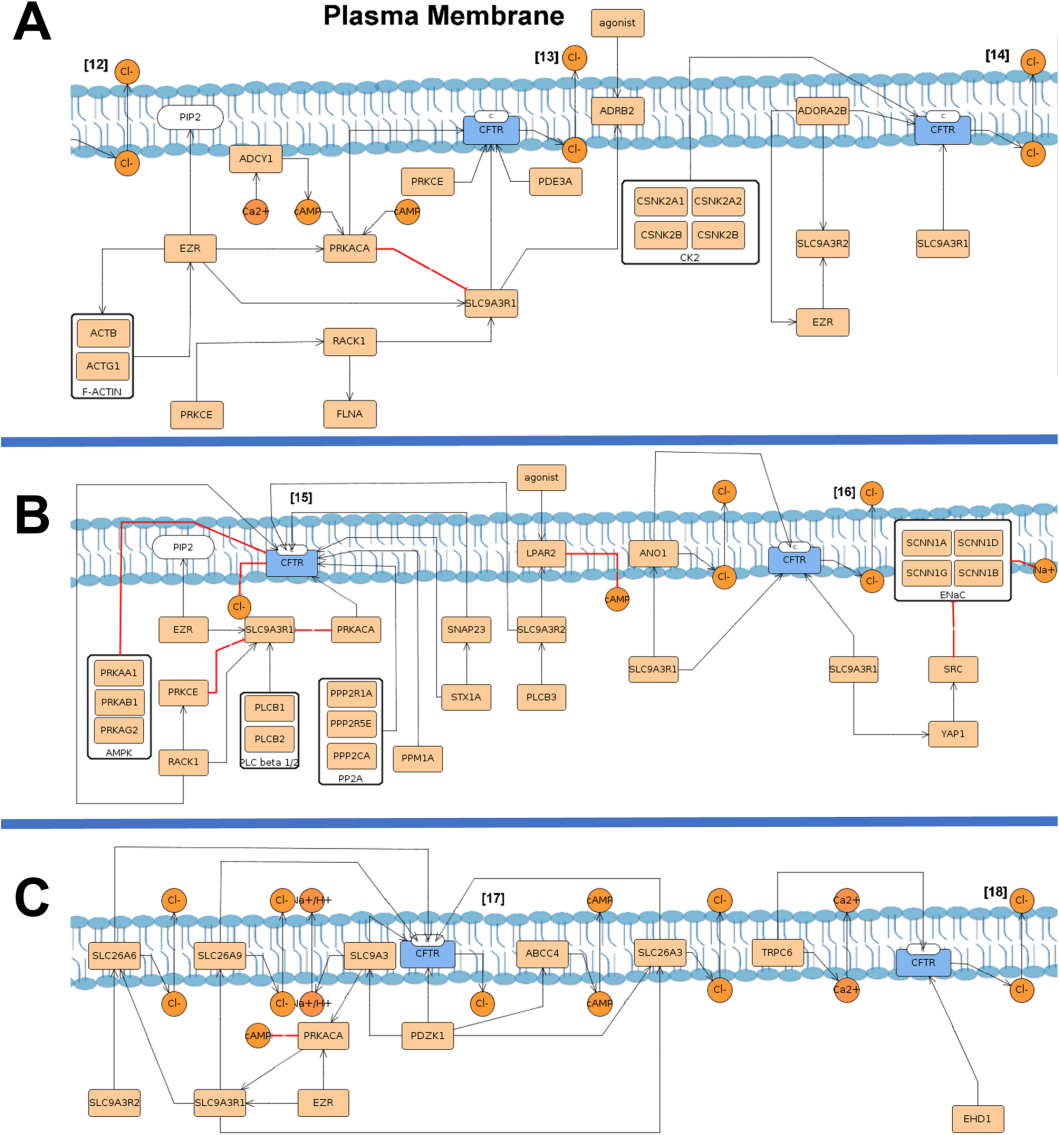
PM pathways in wt-CFTR submap. (**A**) Gating where is possible to see adenosine 3,5-cyclic monophosphate (cAMP) role in Protein Kinase CAMP-Activated Catalytic Subunit Alpha (PRKACA) phosphorylation that leads to CFTR activation and transport of chloride across the membrane. Other proteins are involved such as Adrenoceptor Beta 2 (ADRB2), Protein kinase CK2 (formerly known as casein kinase II), Adenosine A2b Receptor (ADORA2B) and Protein Kinase C Epsilon (PRKCE). (**B**) Shut-down with the action of Lysophosphatidic Acid Receptor 2 (LPAR2), serine/threonine protein kinase complex (AMPK), Phospholipase C beta ½ (PLC beta ½) and Protein phosphatase 2 (PP2A) where the chloride transport is inhibited. (**C**) Transporters and Ion channel regulation, in which the proteins that regulate and are regulated by CFTR at the PM are represented with the interactions detected.

Gating and channel shut down depict proteins involved in *CFTR* activation/inactivation (Fig. 5). *CFTR* is regulated through cAMP and phosphorylated by proteins such as protein kinase A (*PRKACA*) and Protein Kinase C Epsilon (*PRKCE*)^47,48^ (Fig. 5 step [13]).This includes proteins such as β2-adrenergic receptor (*ADRB2*)^49^, A2B receptor (*ADORAB2*)^50^ and adenylyl cyclase I (*ADCY1*)^51^ which participate in cAMP/PKA signalling therefore activating wt-*CFTR* and the transport of chloride.

The Channel shut-down present in both submaps includes dephosphorylation of *CFTR* and the proteins involved in its triggering—such as protein phosphatases, receptors, phospholipases, and others^52,53^.

The transporters and Ion channel regulation module is only present at the wt-*CFTR* submap, and besides directly binding to wt-*CFTR* also allows to visualize PDZ proteins role as intermediates between them, maintaining the proteins in close proximity and leading to changes in their respective functions (Fig. 5B/C, steps [16], [17] and [18]). Proteins such as Solute Carrier Family 26 Member 3 (*SLC26A3*, also known as *DRA*), Solute Carrier Family 26 Member 6 (*SLC26A6*, also known as *PAT1*), Anoctamin 1 (*ANO1*, also known as *TMEM16A*) and epithelial sodium channel (*ENaC*), were included in this module^54–56^.

#### Traffic

In the secretory pathway, traffic is essential for all processes since folding/processing and function to degradation. *CFTR* traffic processes start by Coat Protein complex II (COPII) vesicles, responsible for its transport between ER and Golgi, from where wt- and rF508del-*CFTR* reach the PM (wt-*CFTR* submap, step [9])^43^. rF508del-*CFTR* traffic between ER and Golgi is depicted through the COPII vesicles module with the same mechanisms as wt-*CFTR* (rF508del-*CFTR* submap, step [3])^33,34,57^. Although this information is only supported by high-throughput research articles and with only one paper for each interaction with the mutated protein, it was one of the exceptional cases that were selected to CyFi-MAP given that is explained by the functional context provided in wt-*CFTR*. A list with the exceptions isin the additional information section of the supplementary material.

**Figure 6:**
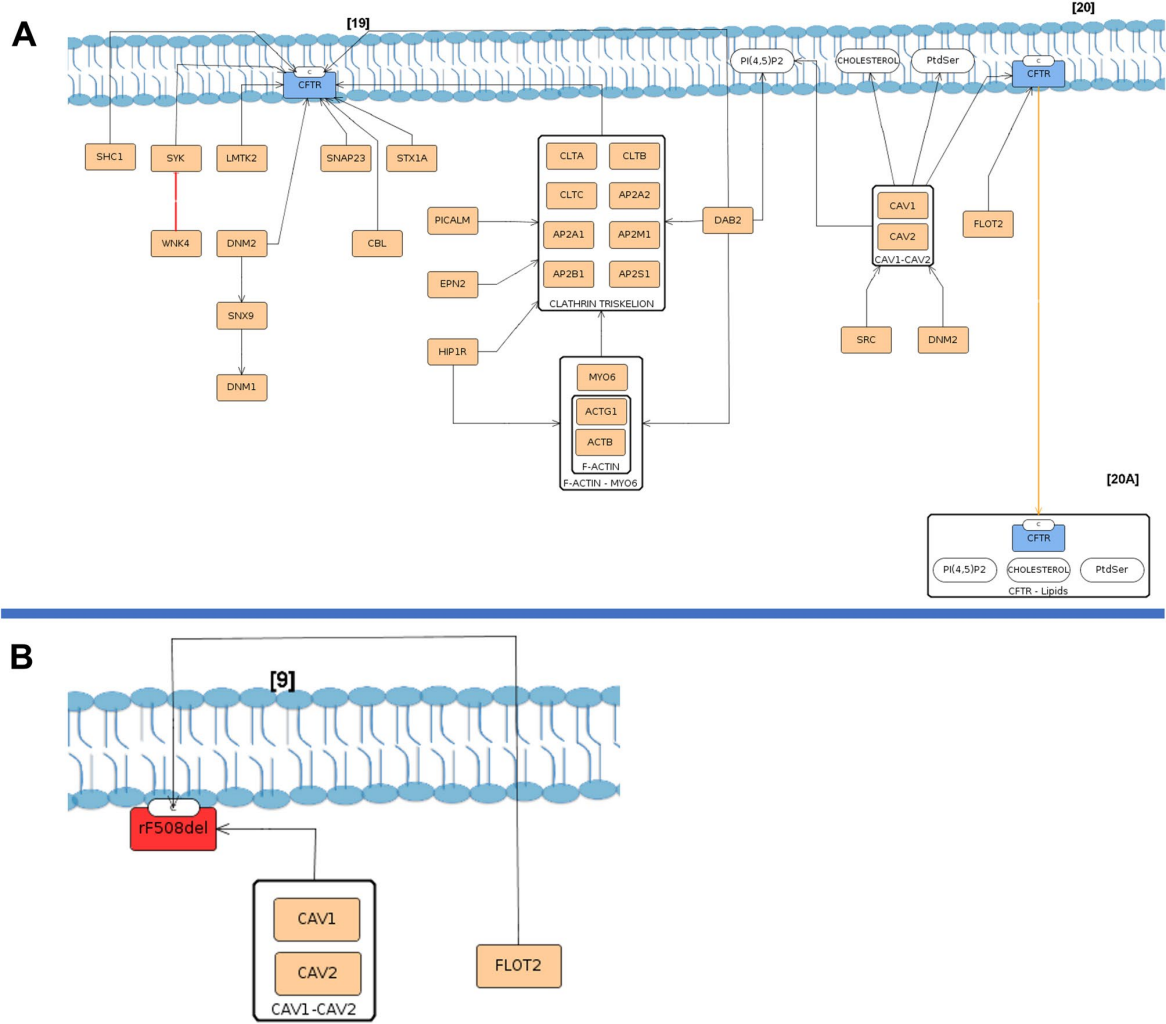
wt-CFTR (**A**) and rF508del-CFTR (**B**) endocytosis mechanisms of internalization from the PM. In the image A two types of internalization of wt-CFTR are present, through clathrin coated vesicles [19] and through caveolae vesicles [20]. As is represented, in the first, several proteins are involved, since the clathrin triskelion complex to cytoskeletal F-actin-MYO6 complex and other proteins assisting the process. In rF508del-CFTR, only the complex Caveolin 1 (CAV1)/ Caveolin 2 (CAV2) was found in this protein endocytosis [9], with assistance of Flotillin 2 (FLOT2).

From this point, the endocytosis module depicts a wt-*CFTR* association with endocytic adaptors undergoing CCV-mediated and caveolae endocytosis (Fig. 6A, steps [19, 20] respectively), whereas in rF508del-*CFTR* only the last one is depicted (Fig. 6B, step [9]).

**Figure 7:**
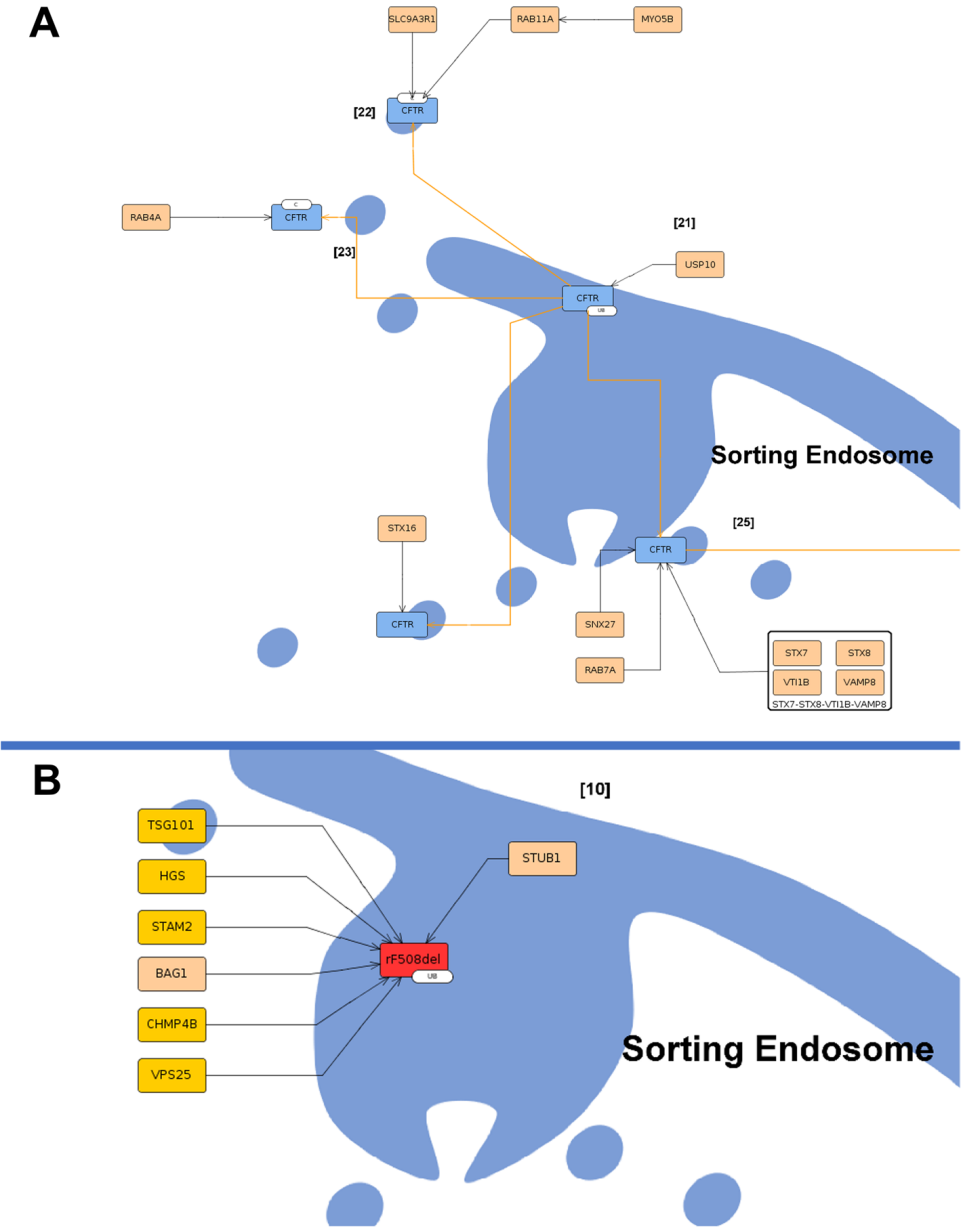
Sorting endosome in wt-CFTR (**A**) and F508del-CFTR (**B**) submaps. In the image (**A**), is possible to see wt-CFTR arriving at the sorting endosome [21] and the possible pathways to follow, either recycling [22], [23] and [24], or degradation [25] in orange lines. Bellow in image (**B**), rF508del-CFTR in red arrives at the endosome [10] in a complex with ubiquitin (represented with Ub) where several proteins target it to degradation directly, representing the low/absence of recycling of this protein. The proteins highlighted in yellow are unique to F508del-CFTR.

*CFTR* is endocytosed and arrives at the sorting endosome from which wt-*CFTR* moves back to the PM, either directly—Recycling Module (Fig. 7A, step [22], [23])—or through the Golgi—Golgi Module (Fig. 7A, step [24])—or to degradation—Degradation Module (Fig. 7A, step [26]). In these processes, several proteins of the Rab family are represented as they are essential for *CFTR* traffic^58^. In the case of rF508del-*CFTR*, at the sorting endosome, it is sent to degradation (Fig. 7B, step [10]).

#### Degradation

During folding and processing, several quality control proteins target misfolded *CFTR* to degradation, therefore, in each of the organelles—ER and Golgi—there is a module called Degradation.

The four ERQC checkpoints can lead towt-*CFTR* degradationat the proteasome. In the case of F508del-*CFTR*, only the first ERQC checkpoint, called chaperone trap, is represented with the binding of four chaperones that strongly attach to the misfolded protein (i.e., Heat Shock Protein Family A (*Hsp70*) Member 4 (*HSPA4*), Heat Shock Protein Family A (*Hsp70*) Member 8 (*HSPA8*), DnaJ Heat Shock Protein Family (*Hsp40*) Member A1 (*DNAJA1*) and DnaJ Heat Shock Protein Family (*Hsp40*) Member B1 (*DNAJB1*)) (F508del-*CFTR* submap, step [2A])^42,59^. Furthermore, the F508del-*CFTR* presents a degradation pathway in contrast to wt-*CFTR*: the sumoylation (Fig. 3B)^60^. At Golgi, *CFTR* degradation depicts its targeting to degradation via the lysosome. In the PM, the rF508del-*CFTR*is targeted to degradation by ubiquitinationas a consequence of the destabilization of the protein (F508del-*CFTR* submap, steps [7], [8] and [9]). There, proteins involved in its degradation at ER attach to itin the PM (i.e., *HSPA8*, *STIP1* Homology And U-Box Containing Protein 1 (*STUB1/CHIP*), Stress-Induced Phosphoprotein 1 (*STIP1*) and others)^61^. At the sorting endosome, in both cells, the protein is targeted to degradation by lysosome/proteasome although with different interactors assisting (i.e., Tumor Susceptibility 101 (*TSG101*), Hepatocyte Growth Factor-Regulated Tyrosine Kinase Substrate (*HGS*), Charged Multivesicular Body Protein 4B (*CHMP4B*), and others) bind uniquely to rF508del-*CFTR* (Fig. 7B, step [10])^62^.

## Discussion

CF is the most common life-threatening autosomal recessive disease in the Caucasian population^63^. Caused by an absent/dysfunctional *CFTR* channel that leads to an impairing balance of ions across the membrane, CF is characterized by affecting several organs, especially the lung^20^. *CFTR* seems to be involved not only in the transport of ions but also in the regulation of other channels, working in a dynamic network that modulates its activity^30^. There have now been more than 30 years of scientific discoveries with new milestones achieved every year in our understanding of intracellular interactions after *CFTR* loss of function that control the progression of this disease. The knowledge obtained over this research has enabled diagnosis and discovery of therapies that increased life expectancy^20^. Yet no final curative treatment has yet been developed for CF disease^64–66^.

The increasing data available on public databases lead to the improvement of tools to filter and extract relevant knowledge required for the discovery of therapeutic targets. With this need, disease maps were developed as a multilayer-readable network that allows representing increasingly complex and extensive information in an easily updatable manner^11^. The visual representation of *CFTR* key processes in a cell can act as a powerful tool to understand and share knowledge. In this work, we built the CyFi-MAP, a manually curated disease map of *CFTR*-related available information, as a resource that permits a deeper understanding and interpretation of the disease mechanisms. CyFi-MAP development is motivated by the absence of resources differentiating between wt-*CFTR* and its variants and has the objective of concentrating on a single free access resource the CF major hallmarks, representing the data scattered across different platforms/research papers in form of pathways and interactions. This tool was designed to be useful for CF scientists as a reference source to analyse previous knowledge and assist in the whole-organism level perspective as well.

### CyFi-MAP included data: wt-CFTR versus F508del-CFTR submap

*CFTR* life cycle can be impacted at several steps, with the most common mutation, F508del-*CFTR*, subjected to ER retention and degradation when not rescued through low temperature or chemical compounds. In fact, rF508del-*CFTR* is characterized by barely reaching the PM under physiological conditions, presenting reduced chloride/bicarbonate transport after being rescued, as well as enhanced endocytosis and degradation, with consequent dysregulation of other PM proteins^53,67^. Due to F508del-*CFTR* incapability to achieve a competent intermediate, the protein becomes trapped in the first steps of ERQC and is mostly targeted for degradation as soon as the polypeptide is synthesized—indicated in CyFi-MAP by the absence of the others ERQC processes^42,43,68,69^. Furthermore, the location and function of *CFTR* at PM are affected in the case of rF508del-*CFTR*, visible by the lack of interactions and by the absence of key processes when compared with wt-*CFTR* in CyFi-MAP. rF508del-*CFTR* in CyFi-MAP lacks proteins involved in the activation of the channel and regulation of other channels and transporters at the PM. This is a consequence of its instability where unique proteins that interact with rF508del-*CFTR*, such as Calpain 1 (*CAPN1*) and Calpain 2 (*CAPN2*), play a role in its destabilization by impairing its binding to PDZ anchor proteins. Besides that, ubiquitination and subsequent targeting to endocytosis and degradation involve additional proteins such as Ring Finger And FYVE Like Domain Containing E3 Ubiquitin Protein Ligase (*RFFL*), *TSG101*, *HGS*, *CHMP4B* and others that prevent the recycling to the PM^62,70,71^.

Some proteins (e.g. *SLC9A3R1*, *PDZK1* and others) involved in several roles along the *CFTR* life cycle (including stabilization, anchoring and function at PM) are present on the map more than once. PDZ proteins are essential elements also that act as intermediates that connect other channels with *CFTR*. Additionally, *PDZK1* is found binding to two *CFTR* proteins, maintaining *CFTR* proteins functioning in close association.

Besides the conventional trafficking, unconventional secretion pathways have been described for membrane proteins such as CFTR and usually involve bypassing the Golgi, a route that was identified through blocking the conventional Golgi-mediated exocytic pathway^72,73^. Pathways such as this, were not included in this version of CyFi-MAP as they are unlikely to represent the cell in its physiological state. Notwithstanding, it can be helpful to provide a broader view of the possible interactions and they may appear in a future version of CyFi-MAP with less stringent criteria for inclusion.

### CyFi-MAP expansion and future work

Given the fact that new data are generated continuously, and CF aspects are yet to be included, the CyFi-Map is constantly developed with support from the community and funding agencies.CyFi-MAP benefits from major features of the MINERVA platform via its online distribution: comments and suggestions from users with regard to changes in the map content (addition, removal, update) can be analysed directly by curators and addressed potentially in the map after further refinements. In this way, users can promote active discussions and knowledge exchange to build an increasingly accurate and continuously updated CF disease map. Notwithstanding, of specific interest as a future direction, is to include in CyFi-MAP the specific steps in *CFTR* processes targeted by compounds, depicting this way the specific target/mechanism where each of them acts. Additionally, we anticipate including a diagram focusing on the process description layer is anticipated of the CF molecular processes (e.g., the N-glycosylation of *CFTR* in ER) in order to provide a deeper understanding of such interactions. Furthermore, the creation of submaps representing other CFTR mutations would be relevant to study the molecular mechanisms affected.

Altogether, CyFi-MAP represents the first stable milestone into a robust and reliable CF knowledge base integrating information on key pathways involved in molecular pathophysiological CF mechanisms, based on curated literature and expert-domain-approval. CyFi-MAP offers an integrative and system-level view of *CFTR* knowledge. CyFi-MAP may support the interpretation of CF progression and may facilitate the development of novel therapeutic targets and strategies. In fact, a better understanding of *CFTR* mechanisms can not only assist in the design of improved therapies for CF but also identify factors that work in other lung diseases, such as COPD or disseminated bronchiectasis. The next steps can also involve the integration of the knowledge acquired using CyFi-MAP as a basis for mathematical models to generate new data through network inference, modelling and creation of new hypotheses to be tested.

## Methods

### CyFi-MAP construction

**Figure 8:**
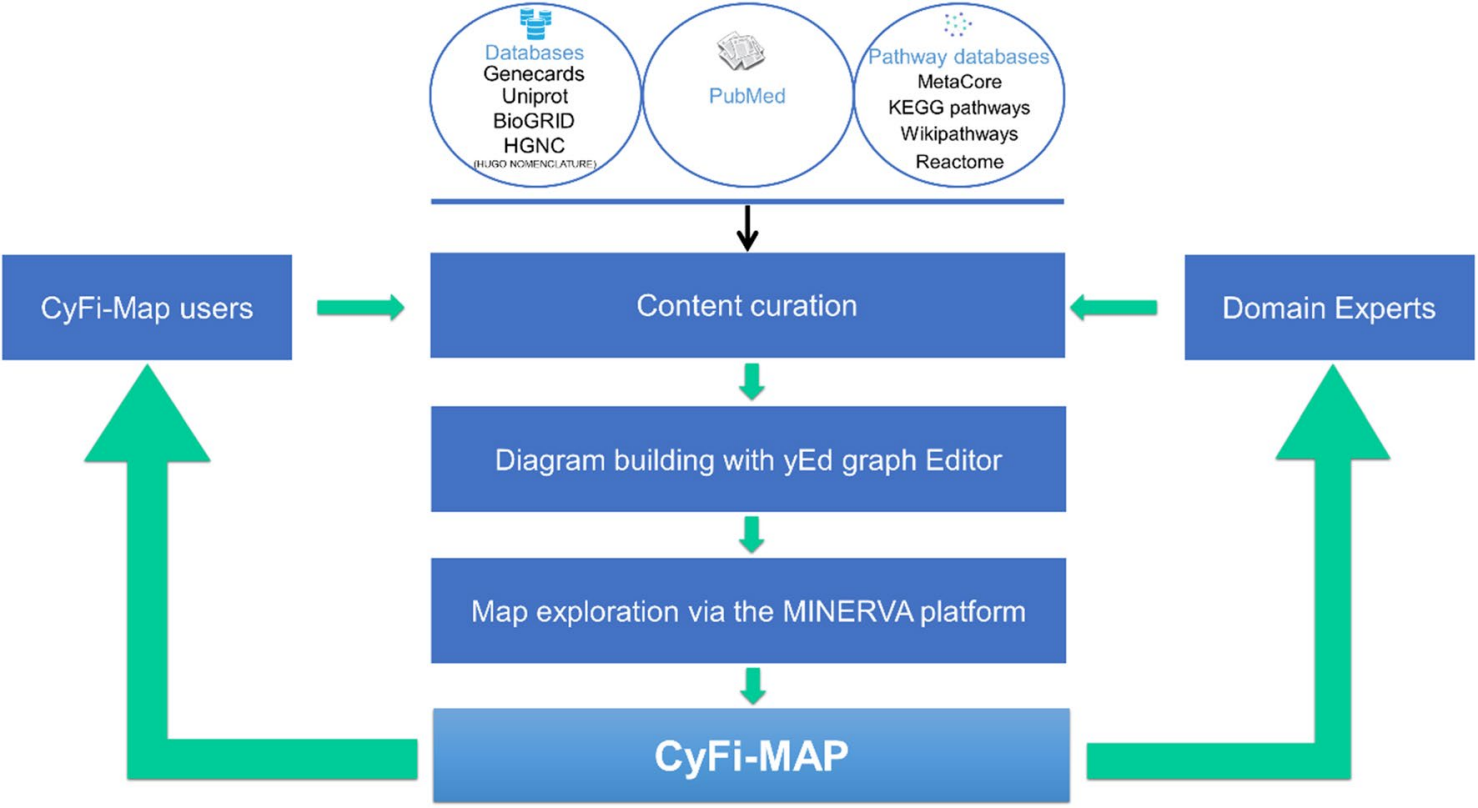
Workflow of CyFi-MAP construction. Starting by the research and curation of data contained in general and CFTR-specific databases and peer-reviewed research papers, CyFi-MAP was constructed based on domain experts’ suggestions and users’ comments. The organization of the curated data required the selection of a general format to be used throughout the platform, which determined the map assembly and content visualization. After the map was built, domain experts were once again consulted, in order to review and provide feedback on the accuracy of the representation of the disease mechanisms, as well as the usability of the platform.

**Figure 9:**
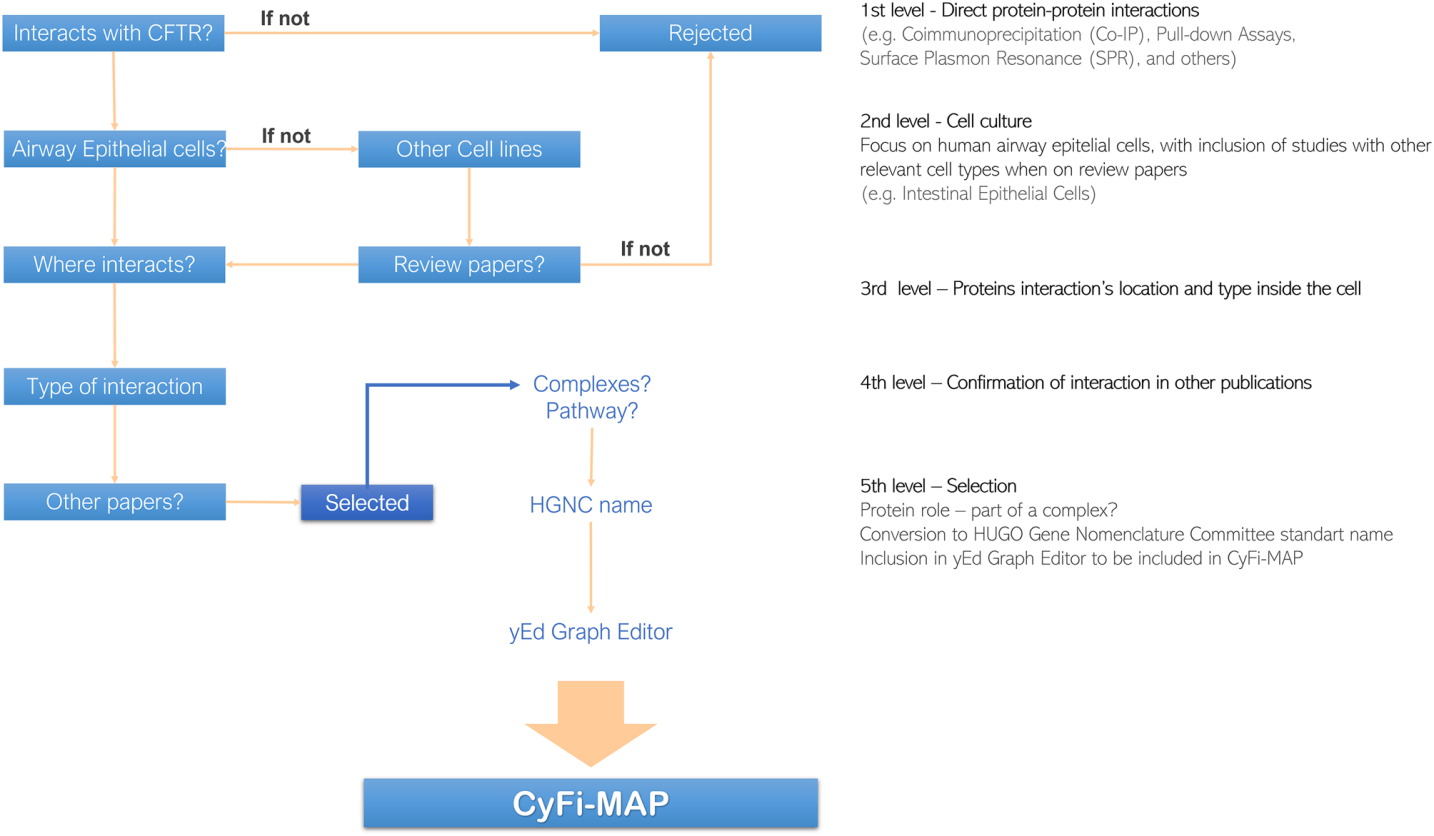
CyFi-MAP curation process. The curation process developed presents 5 levels. 1st level filter the data to studies with proteins that interact directly with CFTR, meaning that only experimental techniques which confirmed a direct interaction were considered such as e.g. Immunoprecipitation, Surface Plasmon Resonance (SRP), and others. The 2nd level is related to the type of cell culture used in these experiments, focusing on human airway epithelial cells, although other cell types were included when described on review publications. In both levels, if the studie do not agree with the criteria is rejected. The 3rd level consist on finding the location of the interaction inside the cell (e.g. ER, Golgi, cytosol, PM) followed by the type of interaction (binding or inhibition). The 4th level confers confidence to the interaction, consisting on the search for publications that support the information. The 5th level confirms information related to the protein after being selected (e.g. does it belong to a complex? Which pathway does it belong to?). The protein is manually added to the yEd Graph Editor used to built CyFi-MAP with the name accordingly to the HGNC nomenclature.

The development of the CyFi-MAP follows the disease map development protocol, using primarily Kondratova et al. and Mazein et al.^11,74^. Specifically, three main steps entail the construction of CyFi-CFTR (Fig. 8):

I. The first step consisted of searching relevant *CFTR*-related information, selecting a total of 297 research papers and more than 1000 reviewed articles. A complete list of publications consulted for the CyFi-MAP development is available in https://cysticfibrosismap.github.io/. The CF disease hallmarks were obtained from peer-reviewed research papers, domain experts’ suggestions and advice, previously documented and validated pathways, and curated up-to-date databases (including Reactome^6^/KEGG^4^/MetaCore from Clarivate Analytics. Please see Content curation subsection for details). This task also involved the analysis of the collected pool of data, followed by the curation of the most relevant *CFTR*-related knowledge (Fig. 9).
II. The second step comprised the effective diagram building, assuring the correct level of detail and the most appropriate and aesthetically output, to guarantee that the resulting map is as readable and user-friendly as possible. The biological mechanisms representation follows the Systems Biology Graphical Notation (SBGN) notation and was built in the yEd Graph Editor using the SBGN Palette (https://yed.yworks.com/support/manual/layout_sbgn.html). The yEd Graph Editor is a freely available graph editor providing functionality to manage large-scale graphs including: (i) features that considerably facilitate the diagram drawing process such as friendly user interface, drawing guides, zooming on the diagram and easy application of specific aesthetics (e.g. same colour for nodes/ edges, curved connectors) to an individual or multiple elements; (ii) algorithms for automatic layout (details on using yEd to automatically layout SBGN-related diagrams are given in e.g.^75^). The yEd Editor also incorporates the SBGN Palette that permits the direct representation of the SBGN-specific elements into the yEd inner GraphML format. After the CyFi-MAP was developed in yEd, we converted it into the SBGN standard format by using the ySBGN converter, (a bi-directional converter between the SBGN and yEd GraphML formats, available at https://github.com/sbgn/ySBGN). Further, the CyFi-Map SBGN diagram was loaded to the MINERVA online platform. The organelle images (developed manually and expert-revised) aim to facilitate visualisation of the mechanisms at the top level; thus, special attention was given to the localization of the interactions in each organelle.
III. The third step in the construction of the CyFi-MAP was the map exploration via the MINERVA platform^37^. In a first approach, the construction focused on the creation of small organelle-specific maps, illustrating *CFTR*-relevant processes on those locations. The maps included *CFTR* interactions covering its intracellular and intraorganellar traffic. Later, these were improved upon by the addition of other, more widespread *CFTR* processes and pathways, which allowed a more effective integration of the existing data. The resulting cell-wide map is expected to be continuously evolving with user input and consistent expert curation. The map is available through the web platform MINERVA that provides interactive and exploratory features.

The current version of CyFi-MAP has been manually curated by CF domain experts. To ensure a continuous updating of this resource, both regular expert verification of new information, as well as regular user input are deemed essential to achieve an accurate representation of current CF data. Constant feedback from cell biologists, biochemists, physiologists and bioinformaticians contributed to a comprehensive representation of the various layers of information.

Inside CyFi-MAP, each process comprises pathways that include proteins (as individual entities or as complexes) and different types of chemical species (ions and lipids) interacting among themselves. Nodes represent entities (i.e., proteins, ions or complexes) and edge colours correspond to processes (i.e., activation, inhibition, synthesis, or in some cases movement of entities inside the cell).

CyFi-MAP currently comprises 618 nodes and 420 edges, with 426 nodes and 307 edges in wt-*CFTR* and 216 nodes and 117 edges in F508del-*CFTR*. In total, entities presented at both submaps are classified into 193proteins, 25complexes, 5ions and 5 simple molecules in wt-*CFTR* and 98 proteins, 12 complexes, 1 ion, and 2 simple molecules in F508del-*CFTR*.

### Content curation

The data used in CyFi-MAP was obtained by manual human search, curation and validation with domain experts in three main sources:

#### Pathway databases

The curation process started by reviewing previous attempts to summarize CF information in these signalling networks. Pathways from MetaCore (Clarivate Analytics), Reactome and KEGG were reviewed in order to analyse the pathway availability for the CF disease^6,76^. Major CF-related pathways were retrieved from these databases and confirmed in the literature for their accuracy.

#### Literature

The main hallmarks of CF were extensively searched in PubMed. As *CFTR* is the protein that plays a central role in the map, direct interactions with it were very carefully selected, following strict criteria. Considering that the lung is the most affected organ, the focus was on human airway epithelial cells studies. The massive number of *CFTR* reviewed articles and studies available were analysed and selected as particularly relevant; results obtained from essays with other relevant cell types (such as intestinal epithelial cells) were also included when validated by review papers, meaning they are accepted by the scientific research community. Priority in the selection process was given to the molecular mechanisms involving protein folding and traffic, as these are the main processes that are impaired in F508del-*CFTR*. Most studies regarding this mutant’s behaviour at the PM resulted from experiments on rF508del-*CFTR*, either chemically or temperature-dependent. The inclusion of information from proteomic studies was dependent on the functional context provided by already documented interactions. Although each direct interaction with *CFTR*on CyFi-MAP was confirmed in a minimum of two papers, some exceptions may apply such as information retrieved from recent articles (2018on) and protein interactions that were part of well-characterized pathways involved in CF referred in more than one peer-review research paper. An example of the last is the interaction between *STX3* with *CFTR*, as only one research paper was found with a physical interaction although is mentioned in peer-review articles^77^ and hence included as one interaction accepted by the scientific community.

#### Databases

Among the web resources used for data gathering, the most significant were GeneCards^78^, Stringdb^79^, Biogrid^80^, UniProt^81^ and HGNC (HUGO Gene Nomenclature Comittee)^82^ which were used to confirm the correct names of proteins/genes, their known function and their interactors. For each protein, the name was checked in HGNC. UniProt and Genecards were used to search for alternative names for the same protein so as to find the correct HGNC designation^78,81,82^. Often, although a protein complex is known to interact/participate in a *CFTR* process, the specific proteins that constitute that complex are not described in the original literature report. Accordingly, proteins reported in the literature to interact with *CFTR* as part of larger complexes were searched for in databases to find the protein components of the complex.

During this step, name disambiguation must be considered in order to find all data related to that protein and also to not repeat proteins. For instance, by looking for syntaxin 5, names such as *Syn5* and *STX5* are also available for the same protein. The same happens for Golgi Associated PDZ And Coiled-Coil Motif Containing protein, known as *GOPC* although other names such as *CAL* and *FIG* are referred to on research papers and used to retrieve as much information as possible.

### Diagram building

CyFi-MAP was built using yEd graph editor (https://www.yworks.com/) using the SBGN Palette, and the data was represented based on the SBGN standard^12^. This notation provides a knowledge representation language used in the illustration of molecular pathways and protein interactions as the standard notation for disease maps^11^. Presenting three languages that provide different types of knowledge illustration allow to adapt on the level of detail intended to be highlighted on the map, including the following layers: Activity Flow to depict interactions with process direction, Process Description detailed specific mechanisms, and Entity Relationships which describe the mechanisms without a sequential process^12^.

CyFi-MAP was implemented following the SBGN Activity Flow, in order to provide a compact, sequential and easy-read format or involving signalling pathways. This language is useful to represent the flow of information in biological sequences/pathways in a way that the information can still be captured for underlying mechanisms of unknown influence^12^.

Each subcellular organelle (ER, Golgi, endosome, etc.) were drawn manually and added to the map as a background image for graphical representation of the different subcellular compartments. Additionally, each interaction provides information on itself. Depending on the selected edge different types of interactions can be found on CyFi-MAP, namely (see Fig. S1 on supplementary material for more details): (1) activation, representing a normal binding, (2) synthesis, when an altered product is released, (3) trafficking, representing movement inside the map, and (4) inhibition, when the interaction inhibits a function.

### Map exploration via the MINERVA platform

CyFi-MAP diagrams are available in the platform MINERVA accessible through GitHub (https://cysticfibrosismap.github.io/). The project description and key processes (shown side-by-side, represented through images to allow the comparison between wt-*CFTR* and F508del-*CFTR* submaps on the CyFi-MAP) are given on the website. Starting at the cell level, it is possible to identify the main differences between the submaps (Fig. 1). This view is relevant in order to compare the cells in presence of the two proteins since wt-*CFTR* is transported across the secretory pathway to the PM and endocytosed to be either degraded or recycled back to the PM, whereas most F508del-*CFTR* is retained at the ER from where it is sent for degradation. This impairment leads to the so-called ‘CF pathogenesis cascade’, which does not occur for wt-*CFTR*. In the cell level view, it is possible to observe these features, allowing the extraction of relevant knowledge.

Additionally, wt-*CFTR* and F508del-*CFTR* submaps were divided into modules, each representing key processes of its life cycle in order to guide the navigation through the map. To compare information in both submaps, images placing the modules folding, stabilization and sorting side-by-side are available. Mutation-specific proteins are highlighted in a different colour to emphasize differences between wild-type and mutated phenotypes.

The interactive web platform MINERVA allows accessing an interactive CyFi-MAP to navigate and explore its molecular networks. This tool provides automated content annotation, direct feedback to content curators and SBGN-compliant format^83^. Navigation in CyFi-MAP is similar to navigation in Google Maps being possible to through MINERVA search elements that are highlighted by markers and also retrieve additional information on each element on the panel on the left side, presenting several identifications names using HGNC and UniProt as sources.

The zoom feature allows a high-level view of the intracellular organelles and a close view inside each providing easier access to the complex and extensive information it contains. Every *CFTR* interaction represented in the CyFi-MAP is validated by PubMed references. The user can curate the data by commenting, given they provide the respective reference as well.

All suggestions will be analysed by curators and CF domain experts to maintain CyFi-MAP quality and accuracy.

The user can contribute to CyFi-MAP by adding comments with questions, corrections or additions to the map. These will be visible to other users and developers. To add a comment to CyFi-MAP during navigation, right-click on the specific location and choose to add a comment. It is possible to choose a specific identity to link the comment, such as the protein or reaction, or to remain ‘general’, which will link the comment to the location the user chooses. The remaining fields allow the user to fill in the name and email in order to facilitate communication with the developers and to clarify any questions that may emerge. Last, there will be a box where comments can be added (Fig. S2). Any supporting information provided will be helpful to incorporate the changes into the map. After sending the comment, it will not be possible to correct it and it will be visible on the map publicly. Details on adding user’s comments in the underlaying MINERVA platform are given at https://minerva.pages.uni.lu/doc/user_manual/v15.0/index/#add-comment.

The CyFi-MAP allow exploring the map with and without seeing the comments provided by the users by clicking on the checkbox Comments above in the map toolbar. These comments will allow the map users to benefit from the domain knowledge and expertise of researchers and to collect valuable information for the research community. All suggestions will be analysed by curators and CF domain experts in agreement with the pre-established curation process to maintain CyFi-MAP quality and accuracy.

## Supplementary Information

Supplementary Information.

## Data Availability

The CyFi-MAP is available at https://cysticfibrosismap.github.io/.
