# Supplementary material for: CyFi-MAP: an interactive pathway-based resource for cystic fibrosis
Source: Sci Rep. 2021 Nov 15;11:22223. doi: 10.1038/s41598-021-01618-3 (PMC8592983; doi:10.1038/s41598-021-01618-3)
Supplement: Supplementary file 1 — Supplementary Information. [file 41598_2021_1618_MOESM1_ESM.docx]

CyFi-MAP - an interactive Pathway-based Resource for Cystic Fibrosis

Catarina Pereira^1,2^, Alexander Mazein^3,4^, Carlos M. Farinha^2^, Michael A Gray^5^, Karl Kunzelman^6^, Marek Ostaszewski^3^, Irina Balaur^3,4^, Margarida D. Amaral^2^, Andre O. Falcao^1,2^

# Supplementary material


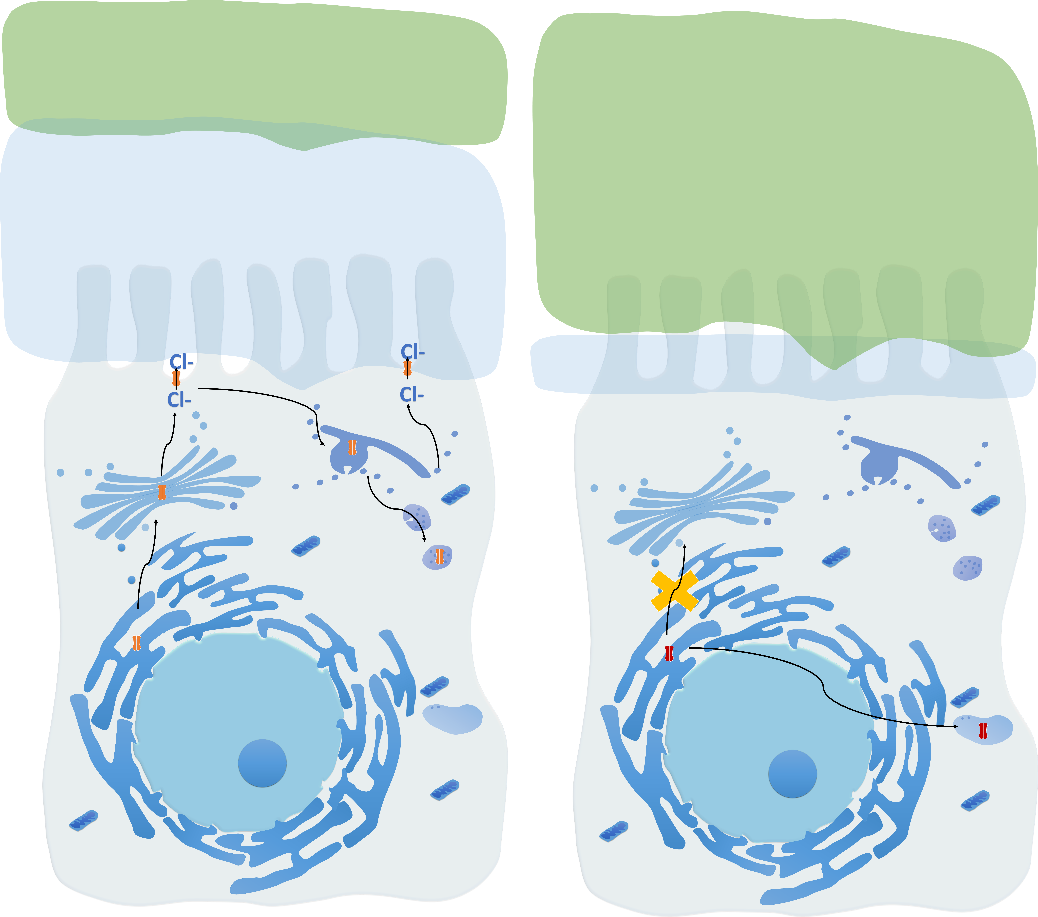


**Figure S1.** wt-CFTR (left) and F508del-CFTR (right) pathways depicted inside an airway shaped lung epithelial ciliated cell. In this scheme it is possible to see the two major consequences of F508del-CFTR mutation: first CFTR PM traffic is disrupted, and this variant is targeted from the ER to premature degradation, hence it does not reach the apical PM where it should transport chloride and bicarbonate. This perturbs the composition and volume of airway surface liquid (ASL) layer and hence the mucus composition, interfering with MCC and leading to mucus plugging, increased inflammation and bacterial infections.

# CyFi-Map processes

**Wt-CFTR**

1. Binding and recruitment of CFTR to the ER membrane
2. Membrane protein integration through SEC61 translocon and addition residues
3. Chaperone binding to assist with folding – may cause CFTR degradation
4. Trimming of one glucose by Glucosidase I action
5. Trimming of one glucose by GANAB action
6. CFTR with one glucose enter CANX cycle to continue folding – may cause CFTR degradation [6A] CFTR re-enter CANX cycle with the addition of one glucose by UGGT1
7. Recognition arginine framed tripeptides (AFTs) motifs – may cause CFTR degradation
8. Recognition ER export motif to pack into coat protein (COP) II-coated vesicles – may cause CFTR degradation
9. CFTR in B form transport from ER to Golgi
10. Regulation of CFTR in C form and transport from Golgi to PM – may cause CFTR degradation

[10A] CFTR degradation by the lysosome

1. CFTR stabilization at PM
2. PDZK1 potentiate CFTR activity by cluster two CFTR molecules
3. CFTR activation through the action of ADRB2 and ADCY1 on PRKACA and PRKCE
4. CFTR activation through the action of CK2 and ADORA2B
5. CFTR shut-down through the action of AMPK, PLC beta ½, PP2A, PPM1A, SNAP23/STX1A and LPAR2.
6. ENaC and ANO1 regulation of CFTR
7. PM channels and transporters regulation of CFTR
8. TRPC6 regulation of CFTR
9. CFTR internalization through clathrin-coated vesicles
10. CFTR internalization through caveolae vesicles [20A] CFTR internalized with species of lipids
11. Deubiquitylation of CFTR through USP10
12. CFTR recycling to PM through RAB11 vesicles
13. CFTR direct recycling to PM through RAB4 vesicles
14. CFTR recycling to Golgi through STX16

[24A] CFTR transport from the sorting endosome to the trans-Golgi

1. CFTR targeting degradation through lysosome
2. CFTR degradation by lysosome and targeting to the proteasome
3. CFTR degradation by the proteasome
4. CFTR targeting to ER degradation through EDEM1 action
5. Chaperone binding to assist CFTR degradation
6. CFTR ubiquitination through STUB1 (CHIP)
7. CFTR polyubiquitination through AMFR
8. CFTR transport from ER to proteasome

**F508del-CFTR**

1. Binding, recruitment and integration of F508del-CFTR in the ER membrane through SEC61 translocon
2. Chaperone binding to assist with folding – main cause F508del-CFTR degradation [2A] Chaperone trap – chaperone association target F508del-CFTR to degradation
3. F508del-CFTR rescued transport from ER to Golgi
4. Regulation of rF508del-CFTR and transport from Golgi to PM – may cause F508del-CFTR degradation

[4A] rF508del-CFTR degradation by lysosome

1. rF508del-CFTR instability at PM through interaction with CAPN1
2. rF508del-CFTR shut-down through action of LPAR2
3. rF508del-CFTR ubiquitination though RFFL
4. rF508del-CFTR polyubiquitination though STUB1
5. rF508del-CFTR internalization through caveolae vesicles
6. rF508del-CFTR targeting to degradation through lysosome
7. rF508del-CFTR degradation by lysosome and targeting to proteasome
8. rF508del-CFTR degradation by proteasome
9. rF508del-CFTR ubiquitination through STUB1
10. Chaperone binding to assist rF508del-CFTR degradation
11. rF508del-CFTR polyubiquitination through RNF5, RNF185 and
12. rF508del-CFTR transport from ER to proteasome with the assistance of DERL1, VCP and SEC61 complex

# CyFi-MAP features

## Table S1 Edges and Entities present at CyFi-MAP according to modules.

|  | Modules | Edges | Entities |
| --- | --- | --- | --- |
| WT-CFTR | Folding | 48 | 62 |
|  | ER degradation | 43 | 46 |
|  | COPII vesicles | 9 | 20 |
|  | Golgi degradation | 19 | 14 |
|  | PM stabilization | 33 | 37 |
|  | Gating | 29 | 34 |
|  | Channel shutdown | 21 | 28 |
|  | Ion channels regulation | 46 | 46 |
|  | Endocytosis | 31 | 44 |
|  | Recycling vesicles | 13 | 12 |
|  | Endosome-Golgi traffic | 2 | 3 |
|  | Sorting endosome degradation | 7 | 19 |

| F508del-CFTR | Folding | 10 | 19 |
| --- | --- | --- | --- |
|  | ER degradation | 53 | 70 |
|  | COPII vesicles | 4 | 14 |
|  | Golgi degradation | 5 | 5 |
|  | PM stabilization | 7 | 8 |
|  | Channel shutdown | 6 | 6 |
|  | Endocytosis | 17 | 22 |
|  | Sorting endosome degradation | 10 | 12 |


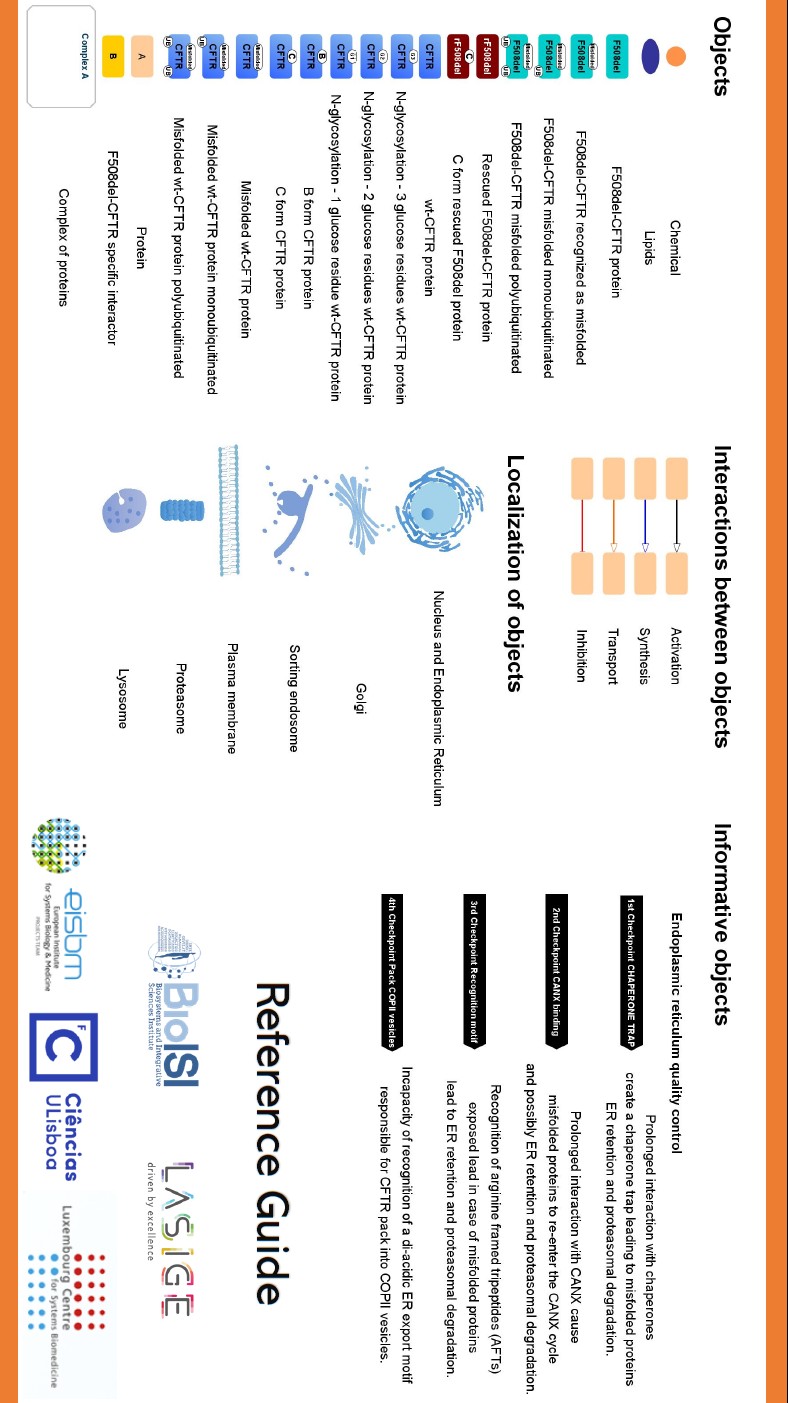


**Figure S2** Reference Guide of CyFi-MAP at GitHub page.

# Additional Information

Exceptions included on CyFi-MAP:

All interactions with only one physical interaction or from proteomic studies were confirmed on review papers.

SLC9A3R2 and LPAR2 interaction on the F508del-CFTR is the only exception that was included based only on review research papers. All other interactions present at least one research paper confirming the physical interaction.


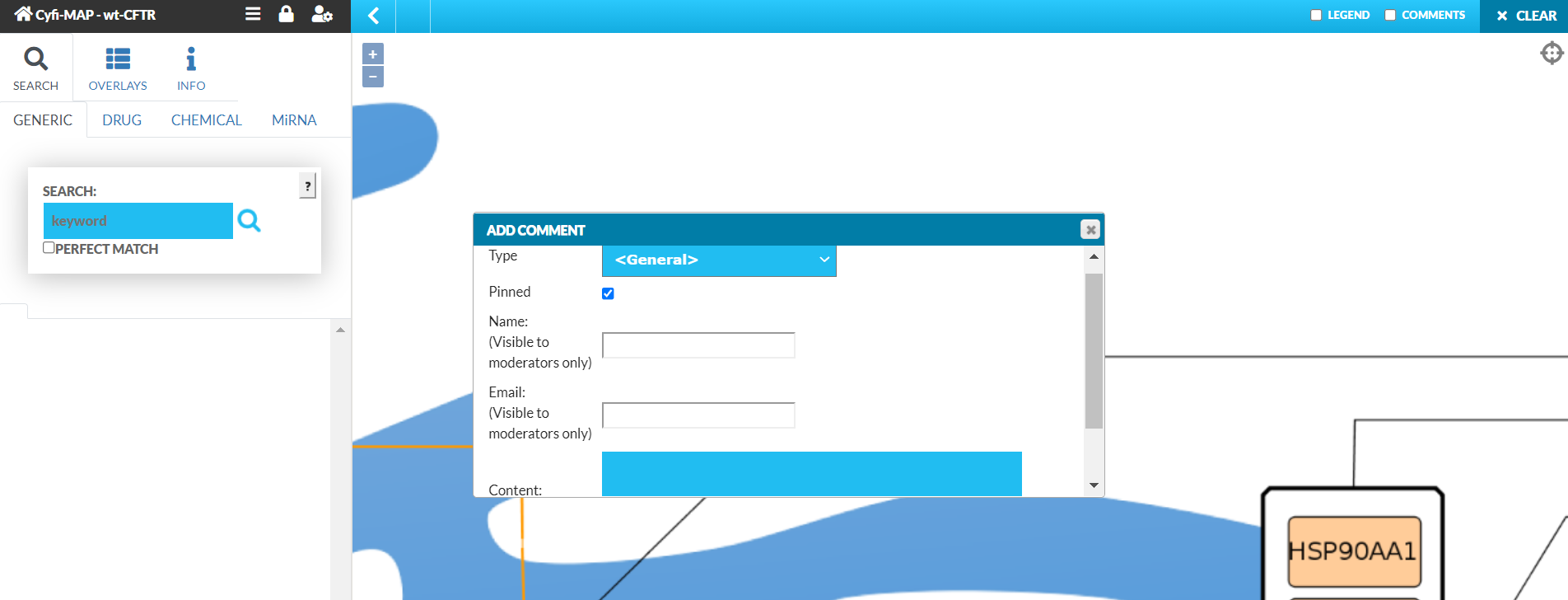


**Figure S3** Directions for researchers to contribute to CyFi-MAP.
